# Vascular Targeting to Increase the Efficiency of Immune Checkpoint Blockade in Cancer

**DOI:** 10.3389/fimmu.2018.03081

**Published:** 2018-12-21

**Authors:** Maria Georganaki, Luuk van Hooren, Anna Dimberg

**Affiliations:** Department of Immunology, Genetics and Pathology, Science for Life Laboratory, Uppsala University, The Rudbeck Laboratory, Uppsala, Sweden

**Keywords:** angiogenesis, cancer, checkpoint blockade, PD-1, PD-L1, CTLA-4, VEGF, endothelial activation

## Abstract

Boosting natural immunity against malignant cells has had a major breakthrough in clinical cancer therapy. This is mainly due to the successful development of immune checkpoint blocking antibodies, which release a break on cytolytic anti-tumor-directed T-lymphocytes. However, immune checkpoint blockade is only effective for a proportion of cancer patients, and a major challenge in the field is to understand and overcome treatment resistance. Immune checkpoint blockade relies on successful trafficking of tumor-targeted T-lymphocytes from the secondary lymphoid organs, through the blood stream and into the tumor tissue. Resistance to therapy is often associated with a low density of T-lymphocytes residing within the tumor tissue prior to treatment. The recruitment of leukocytes to the tumor tissue relies on up-regulation of adhesion molecules and chemokines by the tumor vasculature, which is denoted as endothelial activation. Tumor vessels are often poorly activated due to constitutive pro-angiogenic signaling in the tumor microenvironment, and therefore constitute barriers to efficient leukocyte recruitment. An emerging possibility to enhance the efficiency of cancer immunotherapy is to combine pro-inflammatory drugs with anti-angiogenic therapy, which can enable tumor-targeted T-lymphocytes to access the tumor tissue by relieving endothelial anergy and increasing adhesion molecule expression. This would pave the way for efficient immune checkpoint blockade. Here, we review the current understanding of the biological basis of endothelial anergy within the tumor microenvironment, and discuss the challenges and opportunities of combining vascular targeting with immunotherapeutic drugs as suggested by data from key pre-clinical and clinical studies.

## Introduction

The field of cancer immunotherapy has made significant improvements during the last decade due to the development of new effective means to boost tumor immune responses and achieve long-term remission or even cures in patients that were previously deemed to be untreatable. A major breakthrough was the development of antibodies targeting negative regulators of T-cell activation, termed immune checkpoints. Ipilimumab, an antagonistic antibody targeting cytotoxic T-lymphocyte-associated protein 4 (CTLA-4) improved overall survival in metastatic melanoma patients in 2010 (1). Following the success of anti-CTLA-4 therapy, antibodies targeting programmed cell death protein 1 (PD-1), or its ligand PD-L1, proved to be effective at improving overall survival in a wide variety of cancers (2–7). Importantly, a proportion of patients achieve long-term remission, highlighting the potential of immune checkpoint blockade to induce durable responses (8). The encouraging results of these studies has sparked an interest from the cancer research field and inspired further investigations into targeting of alternative immune checkpoint molecules.

While checkpoint blockade represents a breakthrough in cancer therapy, a majority of cancer patients do not respond and some tumor types appear to be intrinsically resistant. The treatment is designed to boost an ongoing immune response and is inefficient in cases where initial immune activation is lacking, including tumors that are devoid of infiltrating T-cells (3, 9). Development of therapeutic strategies to enhance immune cell recruitment may therefore increase the proportion of patients responding to immune checkpoint blockade. Circulating T-cells are recruited through expression of adhesion molecules and chemokines on the endothelial cells, collectively mediating capture, rolling, and transmigration of leukocytes from the blood stream into the inflamed tissue (10). In many types of cancer, constitutive stimulation by pro-angiogenic factors secreted in the tumor microenvironment renders the vasculature morphologically and functionally abnormal, constituting a barrier to efficient leukocyte recruitment. In this mini-review we summarize phenotypical differences between normal vessels and tumor vessels in mediating leukocyte recruitment, the molecular mechanisms that underlie these functional changes and current efforts to improve immune checkpoint blockade through vascular targeting.

## Immune Checkpoint Blockade Therapy Relies On Efficient T-Lymphocyte Recruitment

Immune checkpoint blockade works through inhibiting negative feedback loops that downregulate T-cell activation following an initial immune response. T-cell activation and T-cell receptor signaling has recently been reviewed in detail (11, 12). T-cells remain naïve until they encounter licensed antigen-presenting cells (APC)s that present the correct peptide antigen on major histocompatibility complex (MHC) molecules together with the appropriate co-stimulatory molecules. T-cell activation requires recognition of the MHC-antigen complex displayed on an APC, engagement of co-stimulatory molecules such as CD28 on the T cell with B7 family members on the APC and stimulation by inflammatory cytokines. In response to T-cell activation, other co-stimulatory molecules such as ICOS and OX40 are expressed, but also molecules that instigate negative feedback loops to prevent over-activation of T-cells. One of those negative feedback molecules is CTLA-4, which competes with CD28 for binding to B7 family members expressed on the surface of APCs (13–16). CTLA-4 is also highly expressed on regulatory T cells, and antibodies targeting CTLA-4 have been suggested to deplete them from the tumor microenvironment through Fc effector functions (17). Although the relative importance of the immune checkpoint and regulatory depletion mechanisms for therapeutic efficacy is still under active debate (16), blocking CTLA-4 in cancer enhances T-cell activation, but can also lead to autoimmune responses.

After activation, which generally occurs in secondary lymphoid organs, T-cells circulate and extravasate through the vasculature at sites of inflammation to locate and kill target cells displaying the cognate peptide antigen on their MHC molecules. At the tumor site, T-cell activity can be hampered by several types of immunosuppression, including engagement of PD-1 expressed on T-cells by its ligand PD-L1 expressed on stromal cells and/or malignant cells (18, 19). Thus, anti-cancer immunity can be enhanced by antibodies that block the PD-1/PD-L1 interaction. Although manipulating T-cell activation status by blocking inhibitory receptors or enhancing co-stimulatory molecules has proven to be efficacious in boosting anti-tumor immune responses, these treatments strictly rely on efficient transport of lymphocytes from the site of T-cell activation to the tumor tissue. It is therefore not surprising that tumors that are not infiltrated by T-cells, and tumors where T-cell infiltration is only observed at the tumor border but not in the core, do not respond well to immune checkpoint blockade (3, 9). Several mechanisms contribute to regulating the inflammatory state, including the mutational landscape of the tumor, expression of chemokines, and checkpoint molecules and recruitment of immunosuppressive cells (20, 21). In cases where an immune response is correctly mounted but where lymphocyte recruitment to the tumor tissue is lacking, pharmacologically altering vascular phenotype to allow efficient leukocyte trafficking may sensitize resistant tumors for immunotherapy.

## Lymphocyte Recruitment Involves Leukocyte/Endothelial Interaction

**Figure 1:**
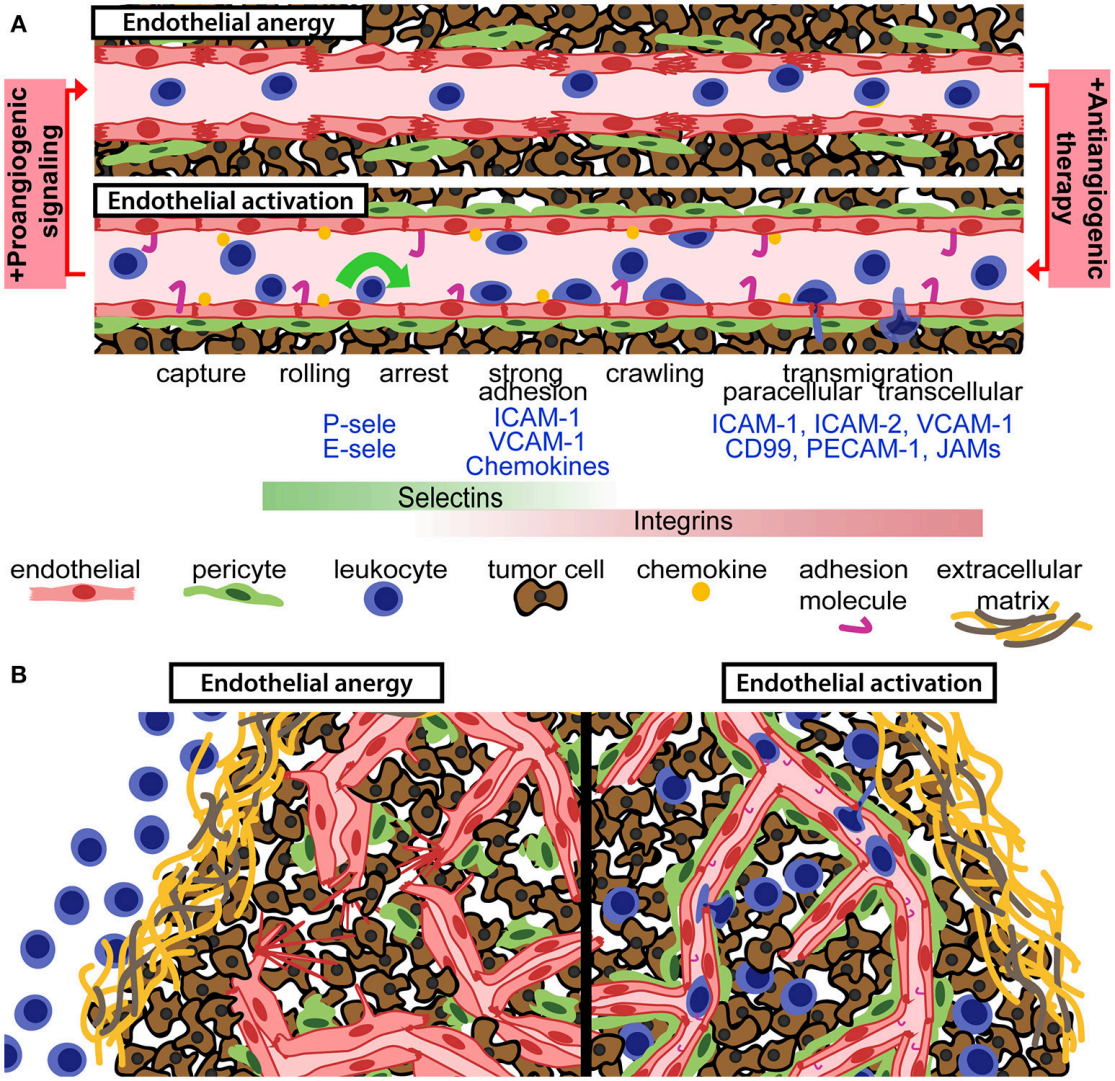
Anti-angiogenic therapy can relieve endothelial anergy, improve vessel function and enhance T-cell infiltration. **(A)** Aberrant pro-angiogenic signaling in the tumor microenvironment gives rise to an anergic endothelium with reduced pericyte coverage, disrupted endothelial cell junctions, and suboptimal activation status. Anti-angiogenic therapy reverts those defects and permits for enhanced leukocyte recruitment, through the leukocyte adhesion cascade. Chemokines and adhesion molecules on the activated endothelial surface allow for leukocyte capture, rolling, arrest, and transendothelial migration into the tumor tissue. **(B)** Aberrant pro-angiogenic signaling in tumors is associated with dysfunctional and anergic tumor vessels, which are not capable of recruiting tumor-targeted leukocytes (left panel). Vascular targeting can relieve endothelial anergy, improve perfusion and increase the recruitment of leukocytes into the tumor microenvironment (right panel).

Leukocyte recruitment by activated endothelial cells and subsequent migration through the vessel wall is mediated by direct molecular interactions between proteins expressed by leukocytes and endothelial cells (Figure 1A). This finely tuned process, known as the leukocyte adhesion cascade, involves leukocyte capture, rolling, adhesion, arrest, and transendothelial migration (10). This is enabled by up-regulation of adhesion molecules and chemokines on the surface of endothelial cells, denoted “endothelial activation.” Leukocyte capture and rolling are mainly mediated by interaction between selectins expressed on endothelial cells (P-selectin and E-selectin) and leukocytes (L-selectin) to carbohydrate ligands including P-selectin glycosylated ligand 1. Firm adhesion of leukocytes is mediated through interaction of leukocyte integrins with endothelial adhesion molecules. For T-lymphocytes, firm adhesion is mainly induced by lymphocyte function-associated antigen (LFA)-1 and very late activation antigen (VLA)-4 binding to endothelial intercellular adhesion molecule (ICAM)-1 and vascular cell adhesion molecule (VCAM)-1, respectively. Activation of integrins via inside-out signaling associated with chemokine stimulation triggers leukocyte arrest to the endothelium (10). Blood flow-derived shear stress contributes to efficient leukocyte capture and integrin activation through mechanical forces (22). Transendothelial migration can occur through either through paracellular or transcellular pathways (10, 23). Finally, leukocytes migrate through the basement membrane and pericyte layer to reach the inflamed tissue (10). Recruitment of lymphocytes to the tumor tissue strictly depends on efficient regulation of molecules required for cell-cell interactions during capture, rolling, adhesion, and transendothelial migration.

## Tumor Angiogenesis Results in Morphologically and Functionally Distinct Vessels

Tumors need access to capillary network to proliferate, and the ability of tumors to stimulate angiogenesis is recognized as one of the hallmarks of cancer (24). Angiogenesis is induced as a result of enhanced growth factor secretion in the tumor microenvironment, shifting the balance from predominantly angiostatic to pro-angiogenic signaling (25). This “angiogenic switch,” observed as a shift from avascular to vascular tumors, can occur in dormant, and slow growing tumors and be associated with tumor progression to higher malignancy grades.

Several mechanisms can trigger neovascularization in tumors, including hypoxia, genetic alterations in tumor cells, expression of cytokines, and growth factorsm and recruitment of bone marrow-derived circulating cells (26–28). When proliferation of malignant cells results in a tumor mass that cannot be sufficiently oxygenated by pre-existing vasculature this leads to hypoxia. Hypoxia-induced stabilization of hypoxia-inducible factor (HIF)-1α triggers up-regulation of its target genes, including several pro-angiogenic genes such as vascular endothelial growth factor (VEGF) (29). VEGF secreted by tumor cells diffuses through the tissue and activates its receptor VEGFR2 expressed on endothelial cells (30). Downstream of VEGFR2 activation, multiple intracellular pathways are induced that regulate cell division, survival, sprouting, and migration of endothelial cells (30). Several other pro-angiogenic growth factors contribute to tumor angiogenesis, including the family of angiopoietins and their cognate receptor TIE-2 and the fibroblast growth factor family (31). Some tumors harbor mutations of the gene coding for the von Hippel-Lindau protein, a crucial member of the ubiquitin ligase complex that degrades HIF-1α (32). These mutations stabilize HIF-1α, allowing expression of pro-angiogenic factors under normoxic conditions. Myeloid cells, including macrophages, neutrophils, and myeloid derived suppressor cells (MDSCs), can also stimulate vessel formation through expression of pro-angiogenic factors and/or matrix metalloproteases that release VEGF from extracellular matrix (33).

Physiological angiogenesis is a well-controlled process that is attenuated when the need for new vessels have been met, but tumor angiogenesis is deregulated and continuous due to excessive expression of pro-angiogenic factors (34). Tumor angiogenesis can give rise to disorganized vessels that are tortuous, dilated and poorly covered by pericytes (35). The tumor vasculature is often leaky due to endothelial junctional defects, blood flow is generally slow and perfusion is irregular (25). Gene expression analyses have shown that tumor vessels differ molecularly from their normal counterparts and have revealed a high level of vessel heterogeneity depending on the resident tumor tissue (36–40). Importantly, tumor vessels can have multiple phenotypes ranging from normal to dysfunctional and the morphology and functionality significantly differ depending on tumor type and anatomical site.

## Tumor Blood Vessels Are Barriers to Efficient Leukocyte Recruitment

Immune cells in the circulation are dependent on the vascular network to reach the tumor and kill malignant cells. However, functional abnormalities of tumor blood vessels represent difficult hurdles for leukocyte recruitment. The architectural defects of tumor vessels limit perfusion and alter sheer stress, and differential protein expression in tumor endothelial cells can dampen the immune response (34, 41–44). Tumor endothelial cell respond inefficiently to pro-inflammatory signaling, and fail to express sufficient levels of molecules involved in the leukocyte capture, adhesion and extravasation process (Figure 1). Downregulation or ineffective clustering of adhesion molecules on tumor endothelial cells limits T-cell infiltration and inhibit anti-tumor immunity (45–47). Reduced expression of adhesion molecules in tumor vessels has been observed in several types of human cancer (48–50). Endothelial activation is generally induced by binding of pro-inflammatory cytokines such as tumor necrosis factor (TNF)α and interleukin (IL)-1 to their endothelial receptors, leading to activation of the transcription factor nuclear factor-κB (NF-κB) and up-regulation of selectins, adhesion molecules and chemokines (51). Pro-inflammatory cytokines are abundantly expressed in many cancers, but pro-angiogenic factors present in the tumor microenvironment can suppress expression of adhesion molecules and chemokines that attract cytolytic T-cells and NK cells such as CXCL10 and CXCL11 (41, 52, 53). VEGF-induced signaling pathways can directly interfere with TNF-α-induced NF-κB activation, globally repressing TNF-α-induced gene expression in endothelial cells (53). Consistent with this, antagonizing VEGFR2 signaling sensitizes endothelial cells to TNF-α (54). However, the interplay between angiogenesis and inflammation is context dependent. TNF-α stimulation synergistically primes endothelial cells for VEGF-induced angiogenesis (55). Notably, VEGF stimulation can induce leukocyte infiltration in some systems, and pathways downstream of VEGF signaling can both induce and repress adhesion molecule expression (56–58). Nitric oxide and molecules such as epidermal growth factor-like domain 7 can also regulate adhesion molecule expression and clustering in tumors (59, 60). Another less studied feature of endothelial regulation of tumor immunity is the selective recruitment of immunosuppressive leukocytes through expression of specific adhesion molecules such as the common lymphatic endothelial and vascular endothelial receptor-1 (CLEVER-1) (41).

In addition to regulating leukocyte entry, tumor endothelial cells can alter the anti-tumor immune response by modulating immune cell activity or viability. This can occur as a response of endothelial cells to tumor-derived growth factors (61). The concept of a “tumor endothelial barrier” refers to molecules expressed on endothelial cells that inhibit promote T-cell arrest. An example of this is tumor endothelial upregulation of FasL in response to tumor-derived VEGF, IL-10 and prostaglandin E2, which has been shown to selectively kill effector CD8 T-cells but not Treg cells (44, 62). Endothelial cells can express several inhibitory molecules including immune checkpoint molecules [PD-L1, T-cell immunoglobulin domain and mucin domain (TIM3), B7-H3 and B7-H4], death receptor-ligands (TNF-related apoptosis-inducing ligand (TRAIL) and secreted immunomodulatory factors (IL-6, prostaglandin E (PGE) 2, IL-10, and TGF-β) (44, 62). The relative importance of endothelial expression of these molecules in immunosuppression and their regulation in tumor vessels need further investigation. Antigen presentation by endothelial cells suggests that they can function as potential antigen presenting cells (63). Whether tumor endothelial cells present antigen and if this is sufficient for activation of T-cells, or alternatively induces T-cell anergy, is still unknown. As discussed below, anti-angiogenic therapies can alleviate endothelial anergy and enhance T-cell recruitment in tumors (53, 64–67). Immunosuppressive molecules expressed on tumor endothelial cells represent new potential targets for novel combination treatments with immunotherapy.

## Successes and Failures of Anti-Angiogenic Therapy

The idea that anti-angiogenic therapy could block tumor progression by depriving the tumor cells of oxygen and nutrients (68) led to intense research efforts and sparked numerous clinical trials. A number of anti-angiogenic drugs have been approved to date, several of which are antibodies or small tyrosine kinase inhibitors that target VEGF/VEGFR signaling (69). The first clinically approved drug was a humanized antibody targeting VEGF named Bevacizumab. Treatment with Bevacizumab slows tumor growth in patients with non-small cell lung and colorectal cancer, though with only a marginal improvement of long-term survival (70, 71). It has also been approved for patients with cervical cancer, glioblastoma, ovarian cancer and renal cell carcinoma (72). In breast, melanoma, pancreatic, and prostate cancer no improvement of overall survival has been observed (73).

Treatment of colorectal cancer patients with Bevacizumab results in an initial response with decreased tumor growth or even regression. However, relapse is common, associated with rapid rebound angiogenesis, and tumor regrowth is often more aggressive than before anti-angiogenic treatment (74). Several mechanisms have been proposed for the resistance to anti-angiogenic treatment, including co-option of normal vessels in the surrounding tissue, recruitment of pro-angiogenic myeloid cells and upregulation of alternative pro-angiogenic factors (25). Notably, anti-angiogenic treatment can increase invasiveness and promote metastasis formation in experimental models of cancer (75, 76). Although metastasis-promoting effects of anti-angiogenic therapy have not been observed in clinical studies, the pre-clinical work has cautioned the field and questioned how anti-angiogenic therapy should best be administrated (77).

## Anti-Angiogenic Therapy can Improve the Effect of Immune Checkpoint Blockade

The importance of a functional vasculature for immune cell recruitment justifies efforts of combining immunotherapy with vascular targeting to improve vessel function and enhance up-regulation of adhesion molecules and chemokines. Inhibition of angiogenic signaling using sub-maximal doses of anti-angiogenic drugs may result in a normalization of vascular function and improve the efficacy of other anti-cancer drugs, as proposed by Jain (78). Anti-angiogenic therapy provides relief of continuous angiogenic signaling, which at sub-maximal doses can result in vessel pruning, maturation, and improved perfusion (69). For cancer immunotherapy, there is an added benefit that anti-angiogenic drugs enhance expression of adhesion molecules and chemokines involved in T-cell recruitment (53, 64–67). Therefore, combining immunotherapy with anti-angiogenic drugs may relieve endothelial anergy and induce lymphocyte infiltration into tumors that prior to treatment were of an immune-excluded phenotype (Figure 1B). Indeed, by combining adoptive T-cell transfer with anti-VEGF therapy in murine melanoma, tumor T-cell infiltration was increased and survival was prolonged (79). An important challenge in this concept is that the dosing of anti-angiogenic drugs is crucial for normalizing vessels and improving T-cell recruitment, and that the optimal dose may differ between patients (80). Nevertheless, the combination of immunotherapy and anti-angiogenic therapy has shown benefit in various therapeutic settings (Table 1).

**Table 1:** Selected studies combining anti-angiogenic therapy with immune checkpoint blockade in preclinical models and clinical trials.

**Anti-angiogenic target**	**Immune checkpoint target**	**Cancer model**	**Survival**	**References**
**PRECLINICAL MODELS**				
VEGF (B20-4.1.1)	PD-L1 (6E11)	SCLC	+ ^**^	(81)
VEGFR2 (DC101)	PD-1 (RMPI-14)	Colon-26 adenocarcinoma	+	(82)
VEGF and ANG2 (Vanucizumab)	PD-1 (RMPI-14)	MMTV-PyMT, RIP1-Tag2, Melanoma, Neuroendocrine	+ ^***^	(83)
VEGFR-1,-2 and−3 (Axitinib)	CTLA-4 (9H10)	Melanoma	+	(84)
VEGFR2^*^ (Sunitinib)	PD-1 (RMPI-14)	Colon cancer	+	(85)
VEGFR2 (DC101)	PD-L1 (10F.9G2)	Pancreatic cancer, breast cancer and glioblastoma	+	(86)
VEGF + ANG2 (10F.9G2 + CVX-241)	PD-L1 (10F.9G2)	Breast cancer	+/-	(87)
**Anti-angiogenic target**	**Immune checkpoint target**	**Cancer type**	**Trial**	**References**
**CLINICAL TRIALS**				
VEGFR-1,-2 and−3 (Axitinib)	PD-1 (Pembrolizumab)	Renal cell cancer	Phase 3	(88)
VEGF (Bevacizumab)	CTLA-4 (Ipilimumab)	Metastatic melanoma	Phase 1	(89) (90) (91)
VEGFR-1,-2 and−3 (Axitinib)	PD-L1 (Avelumab)	Advanced clear-cell renal cell carcinoma	Phase 1b	(92)
VEGFR-1,-2 and−3 (Lenvatinib)	PD-1 (Pembrolizumab)	Renal cell cancer	Retrospective	(93)
VEGF (Bevacizumab)	PD-L1 (Atezolizumab)	Metastatic renal cell carcinoma	Phase 1b	(94)

*Antibody clone or brand name in brackets. SCLC = small-cell lung cancer*,

* broad tyrosine kinase inhibitor,

** *increased T-cell exhaustion*,

*** *increased T-cell numbers and endothelial activation. Ongoing clinical trials are available at www.clinicaltrials.gov and were recently reviewed by Fukumura et al. (95)*.

Drugs targeting VEGF/VEGFR2 signaling have been observed to enhance the response to immune checkpoint antibodies in pre-clinical tumor models. The combination of anti-VEGF and anti-VEGFR2 antibodies prolonged survival in a murine model of adenocarcinoma in combination with PD-1 blockade (82). Similarly, an antibody targeting both VEGF and Angiopoeitin-2 improved responses to PD-1 inhibition in preclinical cancer models (83). The VEGFR inhibitor axitinib combined with anti-CTLA-4, but neither monotherapy, prolonged survival of mice bearing murine melanoma (84). This was associated with increased numbers of CD4^+^ and CD8^+^ T cells in the tumor after the combination treatment. In addition to their effect on vessel phenotype, therapies targeting pro-angiogenic factors can alleviate immunosuppression by directly affecting the immune cells. For example, the tyrosine kinase inhibitor Sunitinib can decrease MDSCs and Tregs (67, 96, 97).

The first phase I clinical trial combining anti-angiogenic therapy with immune checkpoint blockade was a study using Bevacizumab and ipilimumab (anti-CTLA-4). The combination therapy modulated tumor vessel morphology and induced endothelial activation, associated with increased infiltration of dendritic cells and cytotoxic T-cells in melanoma tumors (89, 98). Similarly, combining atezolizumab (anti-PD-L1) with Bevacizumab in patients with metastatic renal cell carcinoma resulted in enhanced trafficking of lymphocytes, and increased cytotoxic T cells (94). Following these promising results, several clinical trials with the same therapeutic rationale have been initiated (95, 98, 99).

## Future Directions beyond Normalization and Endothelial Activation

An emerging concept is that vascular targeting in combination with immune checkpoint blockade may promote tumor immunity by inducing formation of high-endothelial venules (HEV)s. HEVs are specialized vessels found in secondary lymphoid organs that are adapted for lymphocyte trafficking (100). The combination of anti-VEGFR2 antibodies with PD-L1 antibodies induced formation of HEVs and improved T-cell infiltration in the polyoma middle T oncoprotein (PyMT) breast cancer model and the Rip1-Tag2 pancreatic neuroendocrine tumor model (RT2-PNET) (86). Formation of HEVs in glioblastoma models required further stimulation using a lymphotoxin β receptor agonistic antibody, resulting in enhanced T-cell infiltration and reduced tumor growth (86). Vessel normalization in combination with a vascular targeting peptide coupled to LIGHT, a ligand for the lymphotoxin β receptor, induced HEVs and tertiary lymphoid structures in Rip1-Tag5 pancreatic neuroendocrine tumors. Importantly, this therapeutic approach sensitized these tumors to anti-PD-1 and anti-CTLA-4 antibody therapy (101). These studies indicate that beyond normalizing vessels, transforming tumor vessels to HEVs can be of additional benefit in enhancing the response to cancer immunotherapy. Furthermore, HEVs may promote formation of tertiary lymphoid structures which have been associated with a beneficial response to cancer immunotherapy in several types of cancer (100, 102).

Current efforts in vascular targeting aim to improve the efficacy of cancer immunotherapy through inhibition of pro-angiogenic signaling. However, several immunosuppressive molecules that contribute to the tumor endothelial barrier are regulated through alternative pathways, and may be induced secondary to immune activation. This aspect has not yet been sufficiently explored. An increased understanding of the cross-talk between tumor cells, endothelial cells, and immune cells during immune checkpoint blockade therapy may lead to new combinatorial treatment regimens that enhance the abundance of activated T-cells in tumor tissue. This can ultimately increase the proportion of patients that respond to immune checkpoint blockade.

## Author Contributions

All authors listed have made a substantial, direct and intellectual contribution to the work, and approved it for publication.

### Conflict of Interest Statement

The authors declare that the research was conducted in the absence of any commercial or financial relationships that could be construed as a potential conflict of interest.
